# 高效液相色谱-串联质谱法同时测定发酵液中喷司他丁和2'-氨基-2'-脱氧腺苷

**DOI:** 10.3724/SP.J.1123.2020.09018

**Published:** 2021-07-08

**Authors:** Minmin ZHAO, Hongyu ZHANG, Tingting LOU, Kongxiang ZHAO, Suying WANG

**Affiliations:** 1.天津商业大学生物技术与食品科学学院, 天津市食品生物技术重点实验室, 天津 300134; 1. Tianjin Key Laboratory of Food Biotechnology, College of Biotechnology and Food Science, Tianjin University of Commerce, Tianjin 300134, China; 2.天津海关动植物与食品检测中心, 天津 300461; 2. Animal, Plant and Foodstuffs Inspection Center of Tianjin Customs, Tianjin 300461, China

**Keywords:** 高效液相色谱-串联质谱, 喷司他丁, 2'-氨基-2'-脱氧腺苷, 发酵液, high performance liquid chromatography-tandem mass spectrometry (HPLC-MS/MS), pentostatin, 2'-amino-2'-deoxyadenosine, fermentation broth

## Abstract

建立了高效液相色谱-串联质谱(HPLC-MS/MS)同时测定发酵液中喷司他丁和2'-氨基-2'-脱氧腺苷含量的方法。发酵液经高速离心、水溶液稀释、微孔过滤后进行HPLC-MS/MS分析测定。选用Waters Atlantis^®^ T3色谱柱(100 mm×2.1 mm, 5 μm)及其保护柱(5 mm×2.1 mm, 5 μm)进行分离,选择10 mmol/L甲酸铵(含0.1%甲酸)-甲醇(含0.02%甲酸)溶液为流动相进行梯度洗脱,流速为0.3 mL/min,柱温为25 ℃,进样量为10 μL。在电喷雾电离、正离子模式下收集数据,对目标化合物进行定性定量分析。喷司他丁定量离子对为*m/z* 269.17>153.20,碰撞能为11 V, 2'-氨基-2'-脱氧腺苷定量离子对为*m/z* 267.00>136.10,碰撞能为18 V。采用外标法对喷司他丁和2'-氨基-2'-脱氧腺苷定量分析。结果表明,喷司他丁和2'-氨基-2'-脱氧腺苷在1.0~250 μg/L范围内呈现出良好的线性关系,相关系数(*R*^2^)为0.9969~0.9996,相对标准偏差(RSD)为6.51%~8.35%(*n*=8)。在发酵液样品中进行加标水平为1.0、5.0和25 μg/L的添加回收试验(*n*=6),喷司他丁的回收率为97.94%~104.46%, RSD为3.74%~4.88%; 2'-氨基-2'-脱氧腺苷回收率为89.96%~107.21%, RSD为4.81%~13.29%。喷司他丁和2'-氨基-2'-脱氧腺苷的检出限为0.003~0.060 μg/L,定量限为0.010~0.200 μg/L。该文系统性地建立了基于HPLC-MS/MS测定发酵液中喷司他丁和2'-氨基-2'-脱氧腺苷的方法,在实际样品检测中,操作简便,准确度高,灵敏快速,有效克服了基质效应对目标化合物的影响,改善了目标化合物的峰形和稳定性,为从微生物发酵液中检测喷司他丁和2'-氨基-2'-脱氧腺苷提供了方法学基础和借鉴。

## Abstract (en)

An analytical method was established for the simultaneously determination the pentostatin and 2'-amino-2'-deoxyadenosine contents in fermentation broth by high performance liquid chromatography-tandem mass spectrometry (HPLC-MS/MS). After high-speed centrifugation, aqueous solution dilution, vortex shock, and microfiltration, the fermentation broth samples were analyzed by HPLC-MS/MS. The samples were separated on a Waters Atlantis^®^ T3 column (100 mm×2.1 mm, 5 μm) using a gradient elution program with 10 mmol/L ammonium formate (containing 0.1% formic acid) and methanol (containing 0.02% formic acid) as the mobile phases. Moreover, a chromatographic protection column (5 mm×2.1 mm, 5 μm) was added to preserve the column efficiency. The flow rate, column temperature, and injection volume were set at 0.3 mL/min, 25 ℃, and 10 μL, respectively. Qualitative and quantitative analyses of the target compounds were performed using an ESI^+^ source. MS parameters such as the collision energies and tube lens offsets of pentostatin and 2'-amino-2'-deoxyadenosine were optimized. The quantitative ion pairs of pentostatin and 2'-amino-2'-deoxyadenosine were *m/z* 269.17>153.20 and *m/z* 267.00>136.10, respectively; the corresponding collision energies were 11 V and 18 V. The external standard method was used for quantitative analysis. The established method was verified rigorously in terms of the linear range, limit of detection, limit of quantification, recovery rate, and precision. Pentostatin and 2'-amino-2'-deoxyadenosine showed good linear relationships in the range of 1.0-250 μg/L. The correlation coefficients ranged from 0.9969 to 0.9996, and the relative standard deviations (RSDs) ranged from 6.51% to 8.35% (*n*=8). This result indicated good accuracy and exactitude in the detection of the pentostatin and 2'-amino-2'-deoxyadenosine. The recoveries (*n*=6) at three spiked levels (1.0, 5.0, and 25 μg/L) were in the ranges of 97.94%-104.46% and 89.96%-107.21% for the pentostatin and 2'-amino-2'-deoxyadenosine, respectively; the corresponding RSDs were in the ranges of 3.74%-4.88% and 4.81%-13.29%. The limits of detection (LODs, *S/N*≥3) and limits of quantification (LOQs, *S/N*≥10) of the 2'-amino-2'-deoxyadenosine and pentostatin in the fermentation broth were 0.003-0.060 μg/L and 0.010-0.200 μg/L, respectively. The validated experimental method was used for the detection of actual samples, viz. the stored multiple pentostatin-producing mutagenic strains in our laboratory. The HPLC-MS/MS method for the determination of the pentostatin and 2'-amino-2'-deoxyadenosine in fermentation broth offered the advantages of small sampling volume, strong maneuverability, good stability, and high sensitivity. Compared with previously published methods, this systematically established and optimized method significantly reduced the detection time, and matrix effects were well suppressed. Moreover, the peak shape and stability of the target compounds were greatly improved. This method provides a methodological basis and meaningful reference for the detection of the pentostatin and 2'-amino-2'-deoxyadenosine in fermentation broth.

喷司他丁(pentostatin)是一种嘌呤核苷类抗生素,在临床上主要治疗急性T细胞型淋巴细胞白血病^[1,2]^、毛细胞白血病^[3,4]^及慢性淋巴细胞白血病^[5,6]^。喷司他丁在人体中主要作为腺苷脱氨酶抑制剂^[7,8,9]^,可明显抑制腺苷脱氨酶的活性,使癌变细胞中的脱氧腺苷大量积累,抑制癌细胞的核酸合成,从而起到治疗作用。1974年,Peter等^[10,11]^从链霉菌(*Streptomyces antiboticus*)的发酵液中首次分离到喷司他丁,美国食品药品监督管理局(FDA)于1991年正式批准其作为注射剂上市,药品名为Nipent^[12]^。目前,主要通过微生物发酵法获得喷司他丁,而针对发酵液中喷司他丁的检测方法主要包括高效液相色谱法(HPLC)^[13,14,15]^和高效液相色谱-串联质谱法(HPLC-MS/MS)^[16,17,18,19]^。李晓辉^[15]^采用HPLC的方法,以乙酸铵-甲醇作为流动相,使用Agilent Eclipse XDB-C18色谱柱(250 mm×4.6 mm, 5 μm)对发酵液中喷司他丁的检测条件进行了优化,但整个洗脱程序耗时较长(25 min),喷司他丁保留时间为10.1 min,不利于快速检测,而且检测过程伴随着杂质的干扰,分离度低,需要进一步优化。杨鹏等^[16]^采用HPLC-MS/MS,以乙酸铵-甲醇-乙腈为流动相,使用Hypersil ODS2色谱柱(250 mm×4.6 mm, 5 μm),使得喷司他丁保留时间缩短到6 min,但使用该方法连续大量测样后,喷司他丁保留时间出现偏移,峰形变形,色谱柱柱效严重降低。巫攀等^[17,18,19]^应用HPLC-MS/MS对喷司他丁进行检测,选择含0.15%三氟乙酸的甲醇为流动相,采用Shimadzu Inertsil ODS-3反相色谱柱(250 mm×4.6 mm, 5 μm)进行分离,但分析时间仍需30 min以上。以上方法均没有实现喷司他丁的高效检测。随着HPLC-MS/MS技术的愈加成熟,近年来在食品^[20,21]^、药品^[22,23]^等领域被广泛应用,相较HPLC技术,其分离速度、灵敏度等各方面都有显著提升。基于此,本研究主要通过优化色谱柱、流动相及洗脱程序,充分发挥HPLC-MS/MS的检测优势,以缩短喷司他丁的检测时间,增强其分离度和检测精确度,同时实现了喷司他丁伴生产物2'-氨基-2'-脱氧腺苷含量的准确检测。该方法快速、灵敏,准确度高,重复性好,为从微生物发酵液中检测喷司他丁和2'-氨基-2'-脱氧腺苷提供了方法学基础和借鉴。

## 1 实验部分

### 1.1 仪器、试剂与材料

Transcend高效液相色谱-串联TSQ Quantum Ultra质谱装置(美国Thermo Fisher公司); Milli-Q超纯水制备仪(美国Millipore公司);分析天平(瑞士Precisa公司); H1850R高速冷冻离心机(长沙湘仪仪器公司);漩涡振荡器(海门仪器制造公司)。

喷司他丁标准品(纯度≥98%)、2'-氨基-2'-脱氧腺苷标准品(纯度≥98%)、甲酸铵(纯度≥99%)(上海阿拉丁生化科技股份有限公司);甲醇(色谱纯,瑞典Oceanpak公司);本实验所有用水均为Milli-Q超纯水。

### 1.2 标准溶液的配制

分别精确称取0.2510 mg喷司他丁和0.2530 mg 2'-氨基-2'-脱氧腺苷标准品,用超纯水溶解并定容至50 mL,配制成5 mg/L的标准储备液,分装至1.5 mL离心管中,于-80 ℃保存。准确吸取适量喷司他丁和2'-氨基-2'-脱氧腺标准储备液,配制成500 μg/L的混合标准溶液,于-80 ℃保存,备用。

### 1.3 样品前处理

取5 mL发酵液,于4 ℃以10000 r/min离心10 min,取100 μL上清液,用水稀释500倍,经0.22 μm水系滤膜过滤后,进行HPLC-MS/MS检测。

### 1.4 色谱条件

色谱柱:保护柱(5 mm×2.1 mm, 5 μm,美国Waters公司), Atlantis^®^ T3液相色谱柱(100 mm×2.1 mm, 5 μm,美国Waters公司);柱温:25 ℃;流动相A: 10 mmol/L甲酸铵(含0.1%甲酸),流动相B:甲醇(含0.02%甲酸);流速:0.3 mL/min;进样体积:10 μL。梯度洗脱程序见表1。

**表 1:** 喷司他丁和2'-氨基-2'-脱氧腺苷的梯度洗脱程序

Time/min	Flow rate/(mL/min)	φ(A)/%	φ(B)/%
0	0.3	95	5
2	0.3	95	5
10	0.3	5	95
13	0.3	0	100
16	0.3	95	5

A. 10 mmol/L ammonium formate containing 0.1% formic acid; B. methanol containing 0.02% formic acid.

### 1.5 质谱条件

离子源:电喷雾离子(ESI)源;离子源温度:350 ℃;多反应监测(MRM)、正离子扫描模式;喷雾电压:3.5 kV;辅助气压力:103.4 kPa;鞘气压力:310.3 kPa;毛细管温度:300 ℃。母离子、定量离子、离子聚焦透镜电压(tube lens offset)及碰撞能(CE)见表2。

**表 2:** 喷司他丁和2'-氨基-2'-脱氧腺苷的监测离子对、离子聚焦透镜电压和碰撞能

Compound	Parent ion (m/z)	Product ion (m/z)	Tube lens offset/V	CE/ V
Pentostatin	269.17	135.10	78.84	26
	269.17	153.20^*^	78.84	11
2'-Amino-2'-	267.00	114.10	77.78	19
deoxyadenosine	267.00	136.10^*^	77.78	18

* Quantitative ion.

## 2 结果与讨论

### 2.1 质谱条件的优化

实验采用注射泵直接进样的方式将5 mg/L的2种标准品依次注入质谱仪器中,分别在ESI^+^和ESI^-^模式下,进行全扫描,从而确定合适的电离方式,并选择响应较高的离子作为母离子。结果显示,2种标准品均在ESI^+^模式下响应值较高。然后对其离子聚焦透镜电压进行优化。在此基础上,对各母离子进行子离子扫描,选择质谱丰度响应值高且信号稳定的碎片离子作为定量离子,随后对其碰撞能进行优化,以获得更具特异性的质谱方法。优化后得到的喷司他丁和2'-氨基-2'-脱氧腺苷的质谱参数见表2。仪器自动优化功能得到的其他质谱参数详见1.5节。

### 2.2 色谱柱的选择

本实验比较了Waters公司的Atlantis^®^ dC18色谱柱(150 mm×2.1 mm, 5 μm)、Atlantis^®^ C18色谱柱(250 mm×4.6 mm, 5 μm)和Atlantis^®^ T3色谱柱(100 mm×2.1 mm, 5 μm)。在相同的流动相洗脱条件下,观察标准品的峰形和出峰时间。结果显示,当选择Atlantis^®^ C18色谱柱检测时,喷司他丁保留时间在6 min左右,半峰宽略宽,而且随着长时间连续测样后,保留时间出现一定偏移,有严重的拖尾现象,柱效明显降低;当选择Atlantis^®^ dC18色谱柱和Atlantis^®^ T3色谱柱时,喷司他丁在保留时间上相差不大,均在2 min左右,但Atlantis^®^ T3色谱柱在保留和分离强极性化合物上较Atlantis^®^ dC18色谱柱更加优越,而且pH值耐受范围更宽。考虑到发酵液中基质的复杂性,以及实现快速检测的目的,实验最终选用Atlantis^®^ T3色谱柱,并且添加了Waters公司对应的保护柱(5 mm×2.1 mm, 5 μm)以更好地维护色谱柱柱效及寿命。

### 2.3 流动相的选择

本研究在选用Atlantis dC18色谱柱(150 mm×2.1 mm, 5 μm)的条件下,分别考察了以10 mmol/L甲酸铵(含0.1%甲酸)-甲醇(含0.02%甲酸)、10 mmol/L甲酸铵(含0.1%甲酸)-乙腈-甲醇(含0.02%甲酸)作为流动相时,喷司他丁和2'-氨基-2'-脱氧腺苷的保留时间、峰形和响应强度。结果显示,当选择10 mmol/L甲酸铵(含0.1%甲酸)-乙腈-甲醇(含0.02%甲酸)为流动相时,喷司他丁和2'-氨基-2'-脱氧腺苷的峰形不对称,分离度差,均有严重的拖尾现象(见图1a)。当选择10 mmol/L甲酸铵(含0.1%甲酸)-甲醇(含0.02%甲酸)为流动相时,喷司他丁和2'-氨基-2'-脱氧腺可与干扰峰分开,峰宽较小,峰形更好(见图1b)。因此,实验选择10 mmol/L甲酸铵(含0.1%甲酸)-甲醇(含0.02%甲酸)作为流动相。

**图 1:**
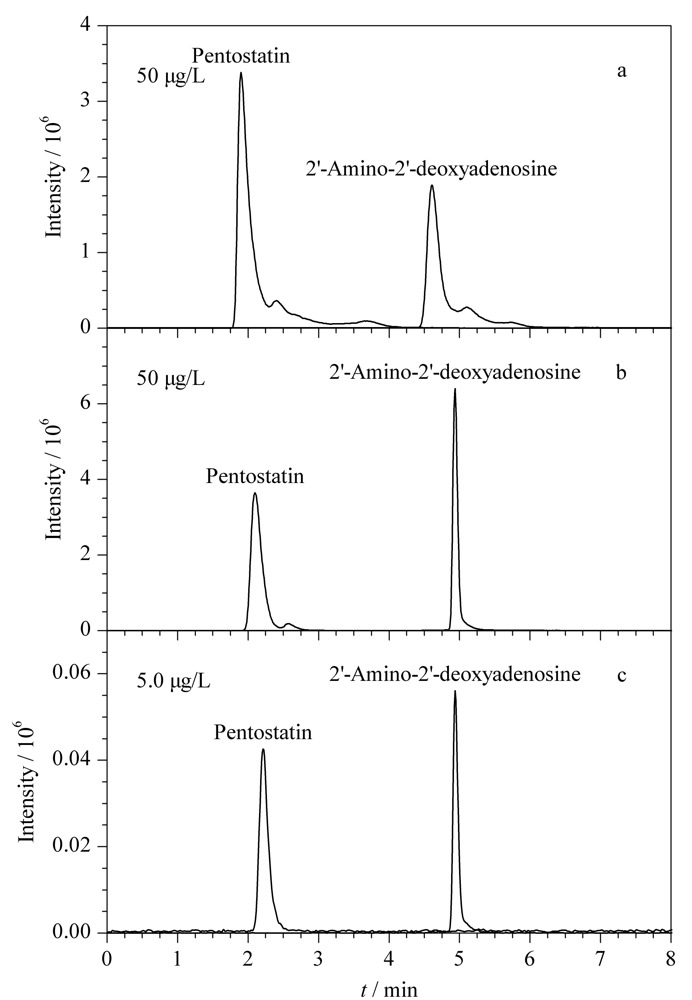
不同色谱条件下喷司他丁和2'-氨基-2'-脱氧腺苷混合标准溶液的色谱图

基于Atlantis^®^ T3色谱柱在保留和分离强极性化合物上的优势,采用Atlantis^®^ T3色谱柱和优化后的流动相10 mmol/L甲酸铵(含0.1%甲酸)-甲醇(含0.02%甲酸),对5.0 μg/L的混合标准溶液进行HPLC-MS/MS测定。结果显示,喷司他丁和2'-氨基-2'-脱氧腺苷均被有效分离,出峰时间稳定,且响应值与峰形良好(见图1c)。

### 2.4 基质效应

基质效应(ME)是指样品中除分析物以外的组分对分析过程存在一定的干扰,从而影响分析结果的准确性。本实验首先采用1.3节样品前处理方法制备空白基质溶液,然后分别以超纯水和空白基质溶液为溶剂,配制质量浓度为100 μg/L的喷司他丁和2-氨基-2-脱氧腺苷混合标准溶液,每个样品各6份,进行HPLC-MS/MS检测,以考察本方法的基质效应(基质效应=基质标准溶液中目标物含量/溶剂标准溶液中目标物含量×100%)^[24]^。

研究表明,喷司他丁和2'-氨基-2'-脱氧腺苷的基质效应分别为103.65%(RSD为2.01%)和107.72%(RSD为2.14%),发酵液中的其他组分会引起一定的基质增强现象,但总体上基质影响较小,比值均接近100%,说明本方法可有效避免基质效应的影响。

### 2.5 方法学考察

2.5.1 线性关系、检出限和定量限

准确吸取500 μg/L喷司他丁和2'-氨基-2'-脱氧腺苷标准储备液,将其配制成1.0、5.0、10、25、50、100、200和250 μg/L的混合标准溶液。在优化后的色谱和质谱条件下制作标准曲线,其中,纵坐标是喷司他丁和2'-氨基-2'-脱氧腺苷的峰面积(*y*),横坐标是与之相对应的质量浓度(*x*, μg/L)。结果显示,2种化合物在1.0~250 μg/L范围内呈现出良好的线性,相关系数(*R*^2^)均大于0.99。将10 μg/L的混合标准溶液重复进样8次,得出峰面积的相对标准偏差(RSD)为6.51%~8.35%(见表3)。

**表 3:** 喷司他丁和2'-氨基-2'-脱氧腺苷的线性范围、线性方程、相关系数、检出限及定量限

Compound	Linear range/ (μg/L)	Linear equation	R^2^	LOD/ (μg/L)	LOQ/ (μg/L)
Pentostatin	1.0-250	y=51806.7x	0.9996	0.060	0.200
2'-Amino-2'-	1.0-250	y=30541.2x	0.9969	0.003	0.010
deoxyadenosine					

*y*: peak area; *x*: mass concentration, μg/L.

本实验分别以3倍和10倍信噪比(*S/N*)的响应值作为喷司他丁和2'-氨基-2'-脱氧腺苷的检出限(LOD)和定量限(LOQ)。检测结果表明,喷司他丁和2'-氨基-2'-脱氧腺苷的检出限为0.003~0.060 μg/L,定量限为0.010~0.200 μg/L。2.5.2 准确度及精密度为了验证该方法的准确度和精密度,综合考虑标准曲线的线性范围和实际检测中喷司他丁和2'-氨基-2'-脱氧腺苷的含量范围,向发酵液样品中添加了3个水平(1.0、5.0和25 μg/L)的混合标准溶液,进行加标回收率试验,每个加标水平做6次重复试验,结果见表4。喷司他丁和2'-氨基-2'-脱氧腺苷的加标回收率分别为97.94%~104.46%和89.96%~107.21%。表明该方法重复性良好,准确度可达到分析要求。

**表 4:** 发酵液样品中喷司他丁和2'-氨基-2'-脱氧腺苷的加标回收率和相对标准偏差(*n*=6)

Compound	Background/ (μg/L)	Added/ (μg/L)	Found/ (μg/L)	Recovery/ %	RSD/ %
Pentostatin	101.769	1.0	102.813	104.46	3.74
	98.669	5.0	103.818	102.98	9.65
	93.649	25	118.134	97.94	4.88
2'-Amino-2'-	229.001	1.0	229.921	92.00	13.29
deoxyadenosine	215.776	5.0	221.136	107.21	4.81
	213.264	25	235.754	89.96	8.08

### 2.6 实际样品检测

从本实验室中随机抽取一株产喷司他丁的突变株,摇瓶发酵后,按照1.3节前处理方法处理发酵液,利用所建立的分析方法对发酵液进行检测。得出该突变株喷司他丁的产量为71.32 mg/L, 2'-氨基-2'-脱氧腺苷的产量为168.60 mg/L(见图2)。从实际样品的色谱图中可明显看出,检测的化合物得到了高效分离,响应高,峰形较好。

**图 2:**
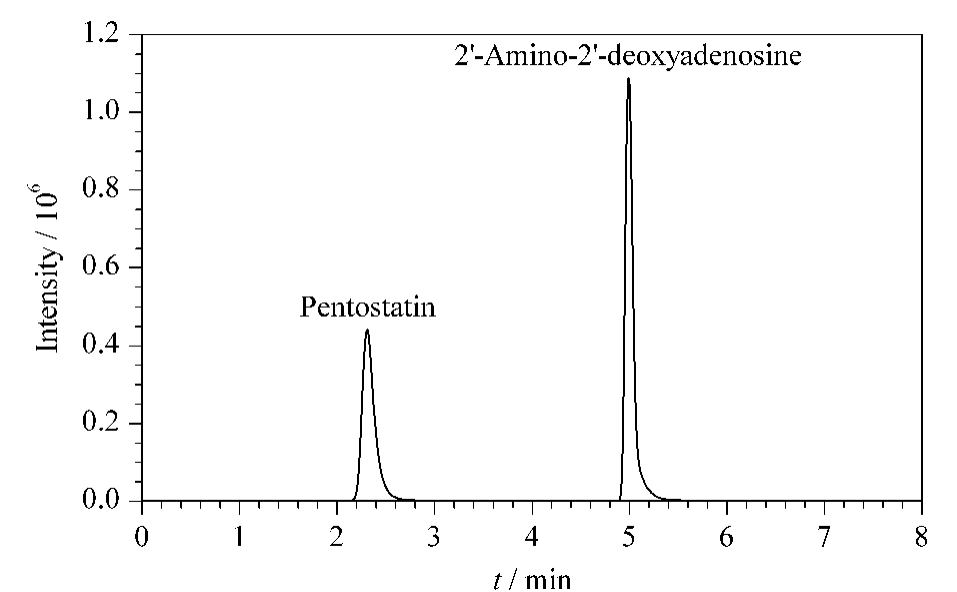
实际样品中喷司他丁(71.32 mg/L)和2'-氨基-2'-脱氧腺苷(168.60 mg/L)的色谱图

## 3 结论

本文建立了高效液相色谱-串联质谱检测发酵液中喷司他丁和2'-氨基-2'-脱氧腺苷的分析方法,并进行了一系列的方法学验证。该方法可操作性强,稳定性高,检测快速,极大地缩短了上机检测时间,有效避免了基质效应对目标化合物的影响,并且为其他发酵液或相似基质中喷司他丁和2'-氨基-2'-脱氧腺苷的定性和定量检测提供了借鉴。

## References

[1] Alfayez M, Thakral B, Jain P, et al. Leukemia Lymphoma, 2019,61(2):1 31566032 10.1080/10428194.2019.1660967 PMC11752443

[2] Jain P, Aoki E, Keating M, et al. Ann Oncol, 2017,28(7):1554 28379307 10.1093/annonc/mdx163 PMC5834082

[3] Kreitman R J, Pastan I. Biomolecules, 2020,10(8):1140 10.3390/biom10081140 PMC7464581 32756468

[4] Alsuliman T, Lassoued K, Belghoul M, et al. Dermatology Ther, 2018,8(1):165 10.1007/s13555-017-0216-z PMC5825321 29196889

[5] Kempin S, Sun Z, Kay N E, et al. Acta Haematol, 2019,142(4):224 31336367 10.1159/000500164 PMC6834875

[6] Kay N E, La P B, Pettinger A M, et al. Expert Rev Hematol, 2018,11(4):337 29460654 10.1080/17474086.2018.1442716

[7] Kay N E, Geyer S M, Call T G, et al. Blood, 2007,109(2):405 17008537 10.1182/blood-2006-07-033274 PMC1785105

[8] Perez J R, Ravandi-Kashani F. Expert Opin Pharmaco, 2020(7):39 10.1080/14656566.2020.1754397 32378970

[9] Sarvaria A, Topp Z, Saven A. Jama Oncol, 2016,2(1):123 26513168 10.1001/jamaoncol.2015.4134

[10] Peter W K, Henry D W, Lange S M, et al. J Heterocyclic Chem, 1974,11(4):641

[11] Henry D W, Peter W K, Ryder A. Ann NY Acad Sci, 1977,284(1):21

[12] Food and Drug Administration (FDA). Drugs@FDA: FDA Approved Drug Products[EB/OL]. (1991-11-10) [2020-08-09]. https://search.fda.gov/search?utf8=%E2%9C%93&affiliate=fda1&query=pentostatin&commit=Search https://search.fda.gov/search?utf8=%E2%9C%93&affiliate=fda1&query=pentostatin&commit=Search

[13] Zhang C Y, Yang S G, Zhang Y, et al. International Infections Diseases (Electronic Edition), 2020(1):13

[14] Zheng X X. Chinese Journal of Antibiotics, 2019,44(12):57

[15] Li X H. [MS Dissertation]. Tianjin: Tianjin University of Science and Technology, 2014

[16] Yang P, Wang Y, Liao Y Y. Chinese Journal of Chromatography, 2010,28(3):316 20549986 10.3724/sp.j.1123.2010.00316

[17] Gao Y J, Xu G D, Wu P. Appl Environ Microb. doi: 10.1128/AEM.00078-17

[18] Wu P. [PhD Dissertation] Wuhan: Wuhan University, 2017

[19] Wu P, Wan D, Xu G D, et al. Cell Chemical Biology, 2017,24(2):171 28111097 10.1016/j.chembiol.2016.12.012

[20] Yang G Y, Guo C T, Xue G, et al. Chinese Journal of Chromatography, 2020,38(12):1388 34213253 10.3724/SP.J.1123.2020.03020

[21] Liu Z Z, Qi P P, He F X, et al. Chinese Journal of Chromatography, 2020,38(12):1396 34213254 10.3724/SP.J.1123.2020.03034

[22] Dai J, Gao L H, Peng F D, et al. Chinese Journal of Chromatography, 2020,38(8):900 34213181 10.3724/SP.J.1123.2019.12025

[23] Yang Y F, Xia Y Y, Wu S, et al. Chinese Journal of Chromatography, 2019,37(12):1291 34213130 10.3724/SP.J.1123.2019.05026

[24] Li J J, Liu G Q. Modern Food Science and Technology, 2019,35(12):294

